# Pityriasis Versicolor—A Narrative Review on the Diagnosis and Management

**DOI:** 10.3390/life13102097

**Published:** 2023-10-22

**Authors:** Nina Łabędź, Cristian Navarrete-Dechent, Honorata Kubisiak-Rzepczyk, Monika Bowszyc-Dmochowska, Anna Pogorzelska-Antkowiak, Paweł Pietkiewicz

**Affiliations:** 1 Department of Dermatology, Paediatric Dermatology and Oncology, Biegański’s Hospital, 91-347 Łódź, Poland; 2 Department of Dermatology, Melanoma and Skin Cancer Unit, Escuela de Medicina, Pontificia Universidad Católica de Chile, Santiago 8331150, Chile; ctnavarr@gmail.com; 3 Department of Dermatology and Venerology, Poznan University of Medical Sciences, 60-356 Poznań, Poland; rzepczykh@ump.edu.pl; 4 Department of Health Sciences, Calisia University, 62-800 Kalisz, Poland; 5 Cutaneous Histopathology and Immunopathology Section, Department of Dermatology, Poznan University of Medical Sciences, 60-356 Poznań, Poland; mbowdmo@ump.edu.pl; 6 EsteDerm Private Dermatology Clinic, 43-100 Tychy, Poland; annapogorzelska03@wp.pl; 7 Dermatology Private Practice, 60-814 Poznań, Poland; 8 Polish Dermatoscopy Group, 61-883 Poznań, Poland

**Keywords:** *Malassezia*, *Pitryrosporum*, yeast, tinea versicolor, fungal infections, dermatoscopy, ultraviolet radiation, microscopy, confocal microscopy, imaging, treatment

## Abstract

This narrative review presents a comprehensive overview of the diagnosis and management of pityriasis versicolor (PV), a common superficial fungal infection caused by the yeast Malassezia. PV is characterised by scaly hypopigmented or hyperpigmented patches, primarily affecting the upper trunk, neck, and upper arms. Regarding commensal interactions, Malassezia utilises nutrient sources without affecting the human host. In cases of pathogenicity, Malassezia can directly harm the host via virulence factors or toxins, or indirectly by triggering damaging host responses. The diagnosis typically relies on recognising characteristic clinical features. Due to the wide variability in its clinical presentation, recognising the differential diagnosis is critical. In this paper, we discuss the clinical differentials, with their dermatoscopic presentation, but also describe a range of helpful diagnostic techniques (microscopy, conventional and ultraviolet-induced fluorescence dermatoscopy, and confocal microscopy). Topical therapies are the primary treatment for PV, encompassing non-specific antifungal agents like sulphur with salicylic acid, selenium sulphide 2.5%, and zinc pyrithione. Additionally, specific topical antifungal medications with either fungicidal or fungistatic properties may also be incorporated into the topical treatment regimen, such as imidazoles, allylamines, and ciclopirox olamine. Systemic therapies might occasionally be used. Patient education and the promotion of good personal hygiene are pivotal to reduce the risk of recurrence. In recurrent cases, particularly during warmer and more humid periods, prolonged prophylaxis with topical agents should be considered.

## 1. Introduction

Pityriasis versicolor (PV), also known as tinea versicolor, is a mild, non-contagious chronic, superficial fungal skin infection caused by lipid-dependent yeast-like fungus *Malassezia* [1]. It manifests as poorly to well-demarcated discoloured or light pink scaly patches, usually affecting the trunk and arms. The disease occurs worldwide but is most prevalent in humid and warm tropical regions. PV tends to be more active in summer seasons [2,3,4]. 

Skin discoloration, associated with enzymatic activity of the yeast and concomitant bacterial colonisation, is transient. However, in many cases, recurrence of the disease may occur despite effective treatment, which adds to the impact on the quality of life of PV patients [5]. Therefore, long-term maintenance treatment is often necessary [1,6,7,8,9].

The diagnosis of PV is often simple. It solely relies on the clinical appearance and hardly ever requires biopsy. However, in clinically ambiguous cases, additional non-invasive work-up (e.g., dermatoscopy, ultraviolet-induced fluorescence dermatoscopy, Wood’s light examination or direct microscopy) may facilitate the diagnostic process [4]. 

In this review, we aim at providing a comprehensive overview on the disease and diagnosis, management, and prophylaxis.

## 2. Aetiology

PV is a fungal infection caused by lipophilic yeast *Malassezia* (previously known as *Pityrosporum*) belonging to the *Basidiomycota* division and the *Malasseziomycetes* class [10]. The *Malassezia* genus, currently comprising 19 species, remains an integral component of the healthy skin microbiome [1,9,11]. *M. furfur*, *M. globosa*, and *M. sympodialis* have been the most frequently isolated in PV [12,13]. On the other hand, *M. restricta* and *M. globosa* are by far the most abundant on human skin, whereas the other species occur less frequently [9,14].

It should be noted that, due to numerous changes in the nomenclature of *Malassezia* and new species constantly added to the genus, it is unclear whether any individual species is responsible for developing PV [9]. Nevertheless, it has been reported that different *Malassezia* species, with different antifungal susceptibility, may coexist in one individual and be responsible for the variability in therapeutic outcome in clinically similar lesions [15]. 

Sebaceous areas of the human skin, including the scalp, face, chest, and upper back, remain a habitat for the *Malassezia* genus due to the abundance of a lipid nutrient source [1,16,17].

## 3. Epidemiology

No sex or ethnic predominance has been reported in PV [12,13,18,19]. PV may affect persons of any age [20]. Nevertheless, it most often develops in adolescents and young adults due to increased sebum production by the sebaceous glands, responsible for the lipid-rich environment, optimal for the yeasts. Higher incidence has been noted in physically active individuals, patients suffering from diabetes and obesity, and immunocompromised individuals. [2,3,4,12,21]. The disease occurs globally, but it is most commonly found in tropical regions and shows a higher incidence during the summer seasons [2,3,4]. The prevalence of PV reaches up to 50% in tropical regions, whereas it is estimated to be around 1–4% and 1% in moderate and cold climates, respectively [1,13,18,19,21,22].

## 4. Pathophysiology

*Malassezia* is a dimorphic yeast-like fungus. It causes PV only in its pathogenic, filamentous (hyphal) form. Factors favouring the conversion from the yeast-like form include a hot and humid environment, hyperhidrosis, the application of oily emollients, using protective face masks, seborrhoea, endocrine and neuropathic disorders, pregnancy, the use of oral contraceptives and corticosteroids, malnutrition, poor general health, and genetic predisposition [2,4,23,24].

The density of *Malassezia* is associated with maintaining skin health, and precisely, *Malassezia* represents the most abundant fungi identified in all areas of the human body except for the feet [25,26]. *Malassezia* spp. constitutes a high proportion of the normal skin microbiome. Thanks to the use of modern diagnostic methods, it was possible to understand the pathogenicity of the fungus, including its participation in commensalism and mutualism [9]. *Malassezia* produces virulence factors and toxins that contribute to its pathogenicity. However, in its commensal relationship, *Malassezia* collects nutrients from the human host without causing negative effects. Furthermore, the concept of mutualism arises when fungal skin colonisation by *Malassezia* provides protection against potentially pathogenic microbes, such as *S. aureus* [27]. In vitro studies demonstrated that *Malassezia*-derived aspartyl proteases have the ability to hydrolyse *S. aureus* protein A, a significant virulence factor that plays a role in immune evasion and biofilm formation [27,28]. These enzymes directly modify the external environment by cleaving extracellular proteins of both the host and other microorganisms. That process plays a pivotal role in facilitating a planktonic cellular state, which could contribute to colonisation. Furthermore, these proteases act as virulence factors, especially in skin with compromised barriers [29,30]. Poh et al. demonstrated, through the use of a 3D acute wound model, that the *Malassezia furfur* secreted aspartyl protease 1 (MfSAP1) has the potential to disrupt wound re-epithelialisation [31].

Understanding the role of *Malassezia* as a direct and indirect cause of PV (via its multifaceted interaction with the skin) is challenging. It is suspected that one of the possible causes for *Malassezia* virulence is the individual genetic susceptibility [32,33]. For example, defects in the skin barrier can alter the composition and/or behaviour of the microbiota, leading to an immune response and skin inflammation. There are two ways in which *Malassezia* interacts with the skin: the first is through direct contact, where specific metabolites of *Malassezia*, for instance, can cause skin irritation. The second way is through indirect contact, where immune or allergic pathways are activated, resulting in skin inflammation [34]. With mild barrier defects, the fungus has a potential to produce hypo- or hyperpigmented patches by interacting with melanocytes. Depigmented lesions develop through enzymatic inhibition of melanocyte tyrosinase activity by azelaic acid produced by the microorganism. The effects of enzymatic depigmentation tend to be more visible in darker phototypes. As a consequence, lipid-like material accumulates in the stratum corneum, acting as a physical sunscreen and protecting from ultraviolet light, further enhancing hypopigmentation. One of the substances suspected of this photo-protective action was pityriacitrin [35], yet its sun-protective properties were proven to be very weak [36]. Hyperpigmented lesions are, in turn, more common in lighter skin tones and they are caused by a hyperaemic inflammatory response elicited by the fungus, increased number of tonofilaments in the granular layer, hyperkeratosis, and the development of abnormally large melanosomes [12]. PV-associated delicate scaling occurs through enzymatic loosening of the horny layer corneocytes with fungal keratinase [37]. Other important virulence factors of *Malassezia* include the production of phospholipase, lipase, acid sphingomyelinases (responsible for sebum lipid degradation), haemolysin, and the ability to produce a biofilm [4,31]. Interestingly, increased fungal lipase activity was associated with a decreased susceptibility to fluconazole, higher haemolytic activity, and biofilm formation potential [38]. It has been suggested that enzymatic patterns and biofilm formation, along with antifungal susceptibility profiles, play a key role in the pathogenicity of *Malassezia* spp. and may explain the involvement of particular species in invasive infections [38]. This phenomenon could also explain the tendency for post-treatment recurrences.

Epidermal hyperproliferation likely stems from the disruption of the skin barrier caused by free fatty acids (FFAs). *Malassezia*, being lipid-dependent fungi, rely on FFAs derived from sebaceous triglycerides. The lipases produced by *Malassezia* release various FFAs from sebum, retaining specific fatty acids while discarding others. These remaining FFAs penetrate the stratum corneum, breaching the skin barrier of the scalp. This skin barrier disruption is evidenced by elevated transepidermal water loss found in patients with dandruff. This phenomenon directly gives rise to the recognisable symptoms of dandruff and seborrheic dermatitis such as itching, flaking, and erythema [32,39].

It has been shown that altering the lipid composition of the growth media for *M. furfur* (CBS 1878 line) drives the volatile compound profiles. These findings highlight how the presence of saturated (e.g., palmitic acid) or unsaturated (e.g., oleic acid) fatty acids in the skin influences the volatile organic compound profile produced by *M. furfur*. This particular species holds significance as it is the sole known species capable of surviving in palmitic acid, the predominant saturated fatty acid in human sebum. The role of volatile organic compounds warrants further investigation due to their potential implications, either beneficial or pathogenic [16].

Fungal MGL_1304 produced by *M. globosa* is a major histamine-releasing antigen, involved in atopic dermatitis and cholinergic urticaria [38,39,40,41,42]. It has been suggested to be involved in PV-associated dermographism (called the “PiMa sign”) [43]. Hyperpigmentation in PV lesions may be associated to some extent also with L-DOPA-dependent melanin and/or black melanin-like pigment synthesis by *Malassezia* observed using the Fontana–Masson stain within the spores and hyphae [44,45]. 

It has been suggested that direct toxic damage to the hair follicle and/or *Malassezia*-induced proapoptotic cytokines may play a role in PV, as *Malassezia* was reported to promote interface dermatitis [46] and upregulate proalopecic interleukins (IL-1a/1b) [47,48,49].

## 5. Clinical Presentation

PV is routinely diagnosed based on clinical observation. It is clinically characterised by hyper- or hypopigmented, poorly to well-delineated, roundish macules covered with delicate translucent scales. Skin lesions may have a tendency towards becoming confluent. The disorder commonly affects seborrheic areas: back and shoulder girdle, neck, chest, and less frequently abdomen, upper arms, and thighs [2,3,4]. Facial involvement is uncommon and has been reported particularly in children [21]. The scalp is a major reservoir of *Malassezia* [50].

Macules and patches occur in various colours including yellow-brown, pale yellow, dark brown, and sometimes even reddish or pink hues. This colour variation is closely associated with the name “versicolor”.

Based on the epidemiological data, the predominance of hyper-/hypopigmented variants in the population seems to depend on ethnic factors as some authors report a high prevalence of hypopigmented lesions [51], whereas others note a high percentage of hyperpigmented ones [2,52]. The fine scales covering the affected skin are non-obvious but may become more apparent when the skin is stretched or scraped (“evoked scale sign”, “Besnier’s sign”, or “coup d’ongle sign”) [53]. In the authors’ experience, if the patient applied the cream prior to the skin examination, the scale may become less apparent or even absent. The disease is often asymptomatic, although some patients may report mild pruritus that may intensify with warmth and high humidity (e.g., after sweating) or if the skin is widely involved [4,52]. Usually, skin discoloration is a major concern for PV patients, especially in darker skin types [4], where it is more common and more apparent. It seems to be cosmetically unsightly and reportedly causes social discomfort, especially when it develops on exposed skin areas [21]. The disappearance of scales confirms therapeutic success [54], yet further weeks to months may be needed for complete repigmentation. PV was reported to induce transient hair thinning or hair loss within the areas affected. This phenomenon seems to be particularly common on the forearms, abdomen, neck, and exclusively in men—in the beard area [49]. 

Some authors have delineated distinct morphological variations of PV, including a variant termed “tinea InVersicolor” or “Inverse PV”. This form is marked by the appearance of lesions in flexural regions such as the axilla, elbow, popliteal fossa, and groin, as well as isolated areas on the extremities [4].

Atrophying PV, another distinct form, typically exhibits multiple, hypopigmented or erythematous/violaceous, round to oval lesions with scaling. These lesions often feature a characteristic depressed appearance, tend to aggregate, and maintain a consistent size. Additionally, these lesions might display a wrinkled surface [4].

Folliculocentric PV predominantly affects the chest and back regions. This form impacts the hair follicles, giving rise to asymptomatic, hypo- or hyperpigmented macules. These macules are exclusively located around the follicles and may occasionally merge to form larger patches [4].

The papular form of PV manifests as numerous, asymptomatic, uniform red-brown papules (measuring 2–3 mm), which could potentially exhibit fine scaling on their surface. These papules are commonly observed on the trunk [4].

The confetti-like subtype is characterised by the presence of the spots resembling confetti, exhibiting a slightly scaly texture. The lesions are typically distributed in a bilateral, symmetrical manner. The development of scaly, guttate, and coalescing hypopigmented patches or plaques is particularly prevalent among African Americans [4].

**Figure 1:**
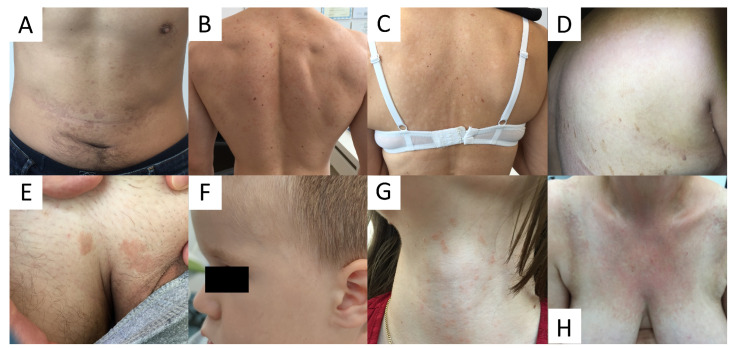
Variability of clinical presentations of pityriasis versicolor (PV). (**A**) Hyperpigmented confluent roundish erythemo-desquamative PV macules over the abdomen in a young man. (**B**) Discrete, achromic confluent PV macules affecting the shoulders and interscapular areas in a young man. (**C**) Depigmented PV spots on the back in middle-aged women. (**D**) Discrete, confetti-like PV depigmented spots on the back of an elderly woman. (**E**) Isolated hyperpigmented “tinea InVersicolor” on the pubis in a young man. (**F**) Roundish, depigmented macules on the face of a young boy. (**G**) Minute reddish scaly spots over the neck in a young female. (**H**) Confluent red scaly macules covering the neckline and presternal area, imitating confluent and reticulated papillomatosis.

Due to high variability in clinical presentation of PV (Figure 1), the differential diagnosis is wide and includes a wide range of hypo- and hyperpigmented disorders (see Table 1).

## 6. Diagnosis

Classical PV rarely requires any work-up other than clinical examination. In challenging, atypical cases, examination through Wood’s lamp, dermatoscopy, ultraviolet-induced fluorescence dermatoscopy, and direct KOH microscopic examination may be of aid. 

### 6.1. Culture

Microbiological culture in PV is not recommended [12]. The process is difficult for the required use of synthetic mycological media enriched with olive oil, and 1–4 weeks of incubation at 32 °C [23,66].

### 6.2. Direct Microscopic Examination

**Figure 2:**
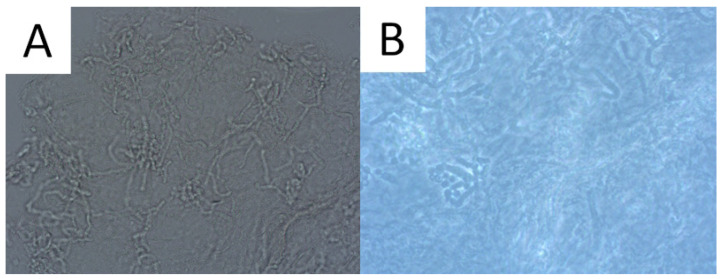
Direct microscopic examination of the skin scrapings reveals yeast cells and hyphal fragments (spaghetti and meatballs appearance), Magnification 200× (**A**). Lactophenol cotton blue stain (LPCB) used to enhance the visibility of hyphae and spores, Magnification 400× (**B**).

Direct microscopic examination is particularly used when the patient presents a typical clinical appearance of PV, but in Wood’s lamp examination, there is no characteristic fluorescence (e.g., after using shampoos with ketoconazole). Skin scrapings are dissolved with a mixture of potassium hydroxide (KOH) and dimethyl sulfoxide (DMSO) and evaluated under light microscopy and phase contrast microscopy. A characteristic appearance of roundish spores and elongated fungal hyphae resembles spaghetti and meatballs (Figure 2) [1,52]. As the highest concentration of fungi is noted at the lesion’s periphery [1,52], we recommend taking the sample from this area to achieve optimal reliability. As adding KOH provides no stain, methylene green, periodic acid–Schiff (PAS; with methylene green or fuchsin), or simply common blue ink can be used for better visualisation [1,67]. A simple scotch-test, involving the adhesion of cellophane foil to the scales, offers an alternative approach for collecting material suitable for staining and subsequent microscopic analysis [68]. Nevertheless, it should be kept in mind that direct microscopic examination with KOH does not provide 100% sensitivity [1,67], and that this method has not been standardised [3]. Thus, supplementary diagnostic tools may be used to obtain a wider context.

### 6.3. Histopathology

**Figure 3:**
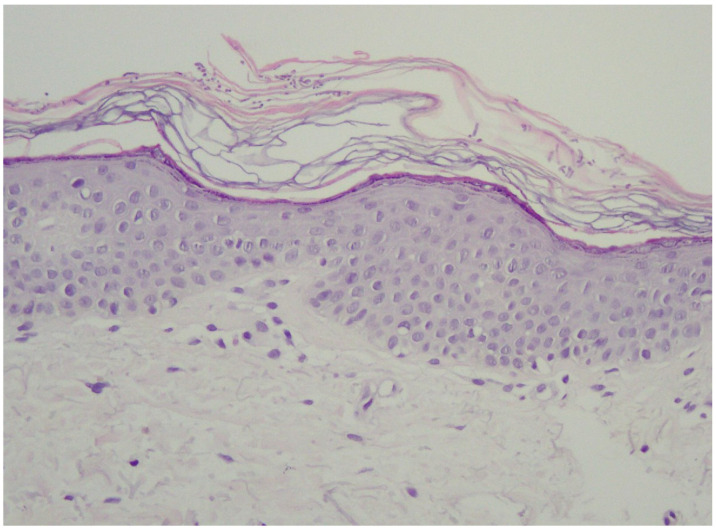
Typical epidermis and dermis with no signs of inflammatory infiltrates or other pathological characteristics. Notably, there is a thickened but loosely arranged stratum corneum. Within this layer, clusters of short longitudinal filaments (resembling hyphae or mycelium) are visible. These filaments occasionally form short branching chains (reminiscent of spaghetti) and round spores resembling yeast cells (meatballs), which can be observed interspersed between the keratin layers. Magnification 20×.

**Figure 4:**
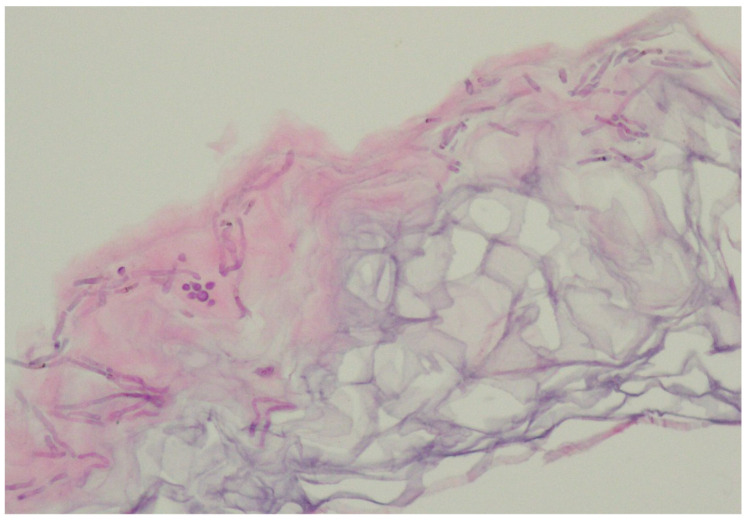
Characteristic basophilic hyphae and spores responsible for spaghetti and meatballs appearance in histopathology. Magnification 40×

In the vast majority of PV cases, skin biopsy remains unnecessary and does not play a role in a daily practice other than for ruling out any of the differential diagnosis [1,22,23]. Thus, the authors advise considering faster and less expensive and less invasive alternatives described below. Histological findings include minimal epidermal changes, some grade of hyperkeratosis, and acanthosis [1,23]. Of note, contrary to what may be appreciated in other superficial mycoses caused by *Trichophyton* or *Microsporon* species, PV features spongiotic changes, whereas the superficial perivascular infiltrate in the dermis is absent or minimal [1,23,69]. Fungal elements can be visualised within the stratum corneum and can be present even in routine haematoxylin–eosin staining as, contrary to other superficial epidermal fungal infections, the spores and hyphal elements are basophilic [69]. Periodic acid–Schiff or Grocott–Gomori methenamine silver stains may further enhance the diagnostic accuracy [1,23] (Figure 3). The classic spaghetti and meatballs appearance may also be appreciated with this method (Figure 4). Hyperpigmented lesions tend to contain more hyphae and spores than the hypopigmented ones [4]. Also, hyperpigmented PV exhibits single, abnormally large melanosomes [70]. On the other hand, hypopigmented lesions feature mild hyperkeratosis and exhibit fewer and smaller melanosomes in the spinous layer when compared to healthy skin [4]. 

### 6.4. Wood’s Light

Wood’s light is an ultraviolet light (UV) emitter (wavelength range: 320–450 nm; peak wavelength: 365 nm). The examination requires a dark environment. UV radiation has the ability to induce excited fluorescence in various substances, called chromophores, including the ones that are present within/on the surface of the skin. If the excited fluorescence belongs to the visible light spectrum, it can be appreciated using visual examination [71,72].

**Figure 5:**
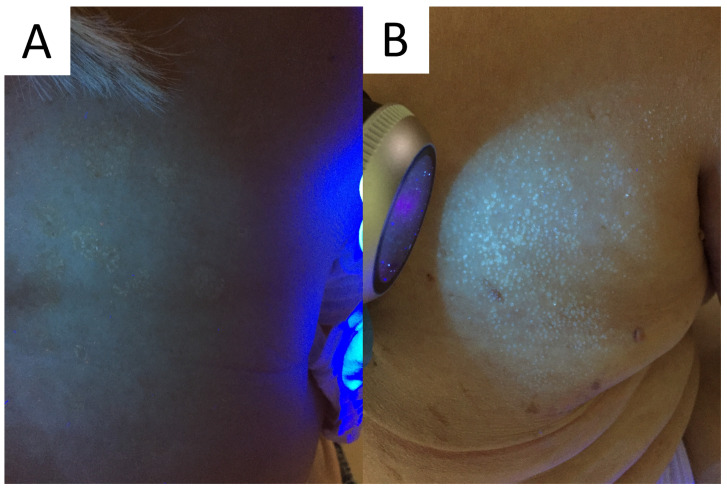
Excited ochre and blue fluorescence seen with Wood’s lamp examination in classic (**A**) and follicular (**B**) forms of pityriasis versicolor, respectively.

The device can be of aid in PV, as UV light may be used to determine the extent of *M. furfur* infection. Typically, a yellow-white or copper-orange excited fluorescence can be observed in an active classical infection and bluish-white fluorescence in the active follicular form (Figure 5) [4,72]. Of note, it has been shown that only one third of the PV lesions display fluorescence with Wood’s lamp [23]. One of the factors seemingly responsible for this number could be washing out the chromophores with a shower/bath prior to the consultation, just as in the case of porphyrin-dependent excited fluorescence in Corynebacterial dermatoses [73].

It should also be kept in mind that cosmetics, sunscreens, topical medications, and other intentionally and unintentionally applied exogenous pigments might potentially interfere with examination, being a potential source of false-positive fluorescence. Nevertheless, this low-cost, fast, and easy-to-apply method has gained great popularity not only in aiding in the diagnostic process and determining the extent of disease, but also in disease monitoring and confirming therapeutic success.

### 6.5. Reflectance Confocal Microscopy

**Figure 6:**
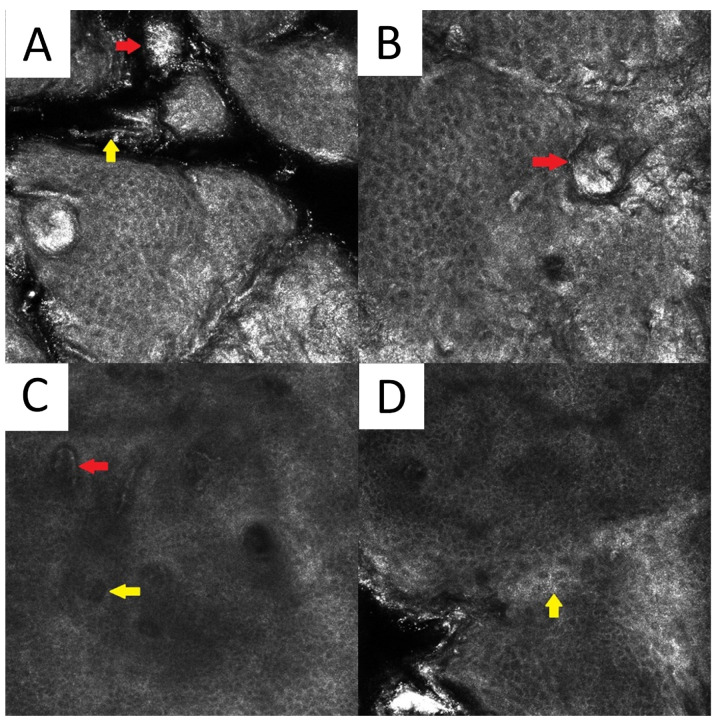
Reflectance confocal microscopy of pityriasis versicolor. Malassezia spores/meatballs (red arrow) and hyphae/spaghetti (yellow arrow) present in the stratum corneum (**A**). Presence of fungi can induce secondary morphological changes: hyperkeratotic plug (red arrow) inside the hair follicle (**B**); elongated vessels (red arrow) and vessels inside papillae (yellow arrow) present at the same level as the lower epidermal layers (**C**); epidermal spongiosis (yellow arrow) (**D**). Magnification 500×

There are scarce data on the reflectance confocal microscopy (RCM) features of PV. Clusters of roundish bright structures and tortuous hyperreflective structures (corresponding to the spaghetti and meatballs appearance seen in conventional direct microscopy) at the level of the horny layer can be seen [74]. Other unspecific findings include the presence of hyperkeratosis, elongated vessels, and spongiotic changes (Figure 6).

### 6.6. Dermatoscopy

**Figure 7:**
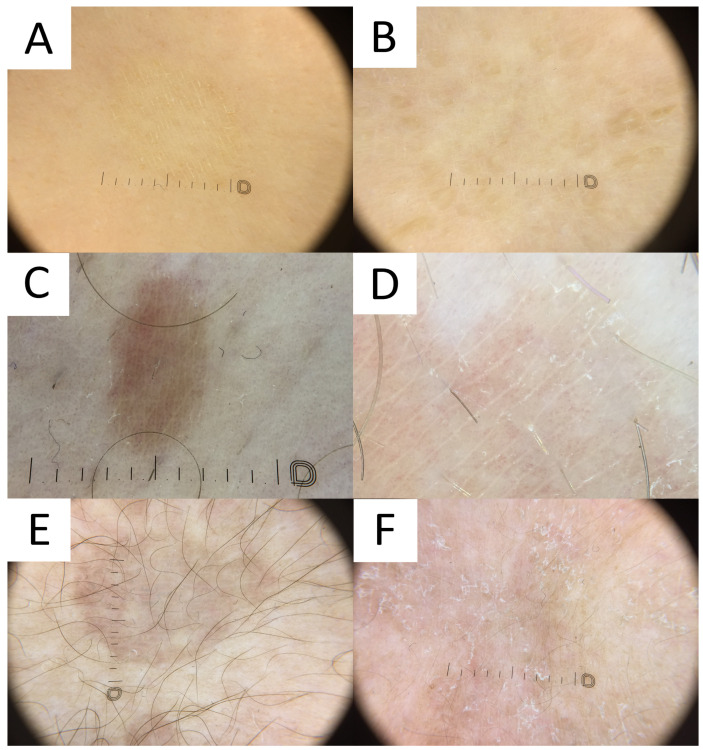
Dermatoscopy in pityriasis versicolor. (**A**) Classic hypopigmented lesion of pityriasis versicolor (PV) displaying typical furrow scaling. (**B**) Follicular hyperpigmented PV displaying small, roundish, folliculocentric tan areas with discrete scaling. (**C**) Hyperpigmented folliculocentric PV lesion exhibiting reddish-tan area with perifollicular, furrow, and peripheral inward scaling. (**D**) Hyperpigmented PV with subtle furrow scales and perifollicular scale. (**E**) Hyperpigmented classic PV lesion with no scale. Peripheral hypopigmented area surrounding the lighter centre (“contrast halo sign”) can be appreciated. (**F**) Hypopigmented PV exhibits diffuse scaling and peripheral hyperpigmented areas (“contrast halo sign”).

PV is usually diagnosed based on clinical features related to the characteristic appearance and distribution of skin lesions. However, cases with atypical distribution or morphology may pose a diagnostic challenge and require differentiating with various pigmentation disorders. Non-contact polarised dermatoscopy is a gold standard in inflammoscopy (Figure 7) [75]. Both hypopigmented and hyperpigmented PV lesions display particular clues [2]. PV patches typically display relatively well-defined depigmented (hypopigmented variant), tan, or red-brownish (hyperpigmented variant) structureless areas covered with fine scales. In darker skin types, these areas display physiological hyper- or hypopigmented reticular lines [52]. It has been noted that in both hypopigmented and hyperpigmented lesions, the pigmentation is usually nonuniform [2]. The scales typically occur in the skin furrows and around the hair follicle openings, due to the higher humidity of these areas [75]. Scaling is more frequently observed in hypopigmented lesions, while furrowed scaling is more common in dermatoscopy of hyperpigmented lesions [2], possibly due to the better contrast between the bright scale and darker background. Of notice, the scale can be completely removed with alcohol solution [76]. Other clues observed in PV may include the peripheral area of hyperpigmentation surrounding the central hypopigmented area or the peripheral area of hypopigmentation surrounding the hyperpigmented centre (described by the authors as a “contrast halo sign”), peripheral hyper/hypopigmented satellite globules, folliculocentricity of the lesions, and hypopigmentation of the affected hair follicle opening (due to fungal invasion) [2,51,52].

### 6.7. Ultraviolet-Induced Fluorescence Dermatoscopy

Ultraviolet-induced fluorescence dermatoscopy (UVFD) is a novel dermatoscopic method utilising UV light. Similarly to Wood’s lamp, UVFD produces colourful images due to the excited fluorescence emitted by the chromophores [71,77]. The main sources of luminescence in PV are blue background elastin and collagen [71,78], bright blue keratin [78], yellow fungal pityrialactone [79], and orange, pink-to-coral-red porphyrins (esp. coproporphyrin III and protoporphyrin IX) [71,78,80].

**Figure 8:**
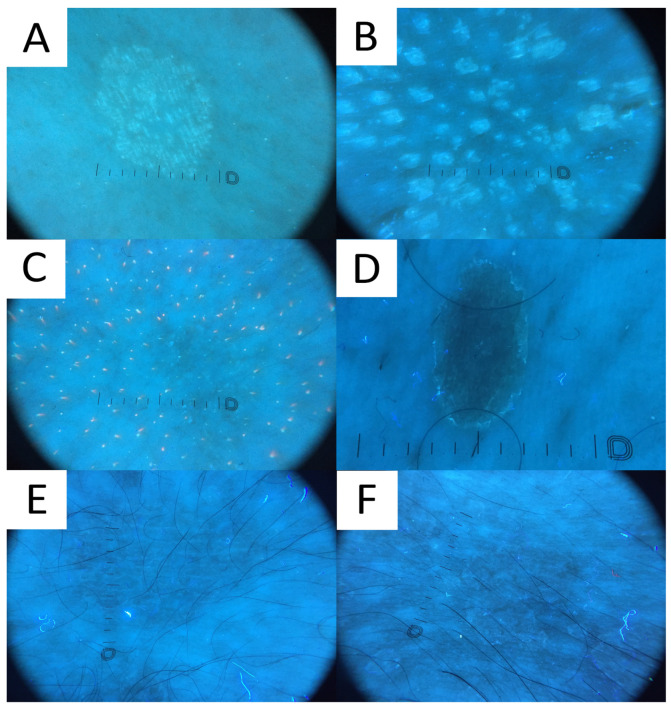
Ultraviolet-induced fluorescence dermatoscopy (UVFD) of pityriasis versicolor (PV). (**A**) Light greenish excited fluorescence is emitted by fungal chromophore (pityrialactone), which results in emergence of single- or double-edged furrow scale and perifollicular scaling, which seem to be better seen in hypopigmented PV lesions. The image matches Figure 7A. (**B**) Light greenish perifollicular scale can be appreciated in active follicular PV strongly enhancing the diagnostic clues of scale. The figure matches Figure 7B. (**C**) UVFD in a case of achromic PV at seborrheic site displays “blackout areas”—areas deprived of background pink-orange porphyrin fluorescence, possibly due to antibacterial properties of azelaic acid produced by *Malassezia spp*. (**D**) Dark greenish fluorescence of folliculocentric hyperpigmented PV lesion. Subtle peripheral free edge of scale is seen better with UVFD. The figure matches Figure 7C (**E**) A case of hyperpigmented PV lesion showing no fluorescence in UVFD. The figure matches Figure 7D. (**F**) “UVFD contrast halo sign” showing dark curvilinear border enclosing hypopigmented PV.

Even though no literature data exist on UVFD clues to PV, hypopigmented lesions can be characterised by light greenish structureless areas, whereas hyperpigmented lesions feature dark greenish structureless areas (PP, personal observations) (Figure 8). In seborrheic areas, the affected sites become deprived of the background red follicular fluorescence of *Cutibacteria* spp., likely due to the antiseptic properties of azelaic acid produced by *Malassezia* spp. (PP, personal observations). UVFD enhances the visibility of the light greenish scale in the skin furrows (that may display a double edge) and light greenish perifollicular scaling even in non-obvious lesions (revealing their folliculotropic nature) (PP, personal observations). Some of the lesions might be surrounded by the darker area visible only with this method (a UVFD equivalent of “contrast halo sign”) (PP, personal observations). The aforementioned observations require confirmation with a larger study group, optimally in an international study representing different ethnic groups. As the scaling and fluorescence are likely to disappear with the elimination of the *Malassezia* fungus and the background cutibacterial follicular fluorescence is likely to reappear with the cessation of azelaic acid production, UVFD can be utilised not only in the diagnostic process, but also in disease monitoring and confirmation of successful treatment. Nevertheless, up until now, there have been no observations on specific time frames when these phenomena occur. In one of the author’s experiences (PP), patients are mesmerised by the luminescent neon-like images shown on the monitor/smartphone screen. Moreover, the instant confirmation of the fungal infection becomes more perceptible to the patients, who seem to adhere stronger to the treatment.

## 7. Treatment

Topical therapy is the treatment of choice for PV, including both specific and non-specific antifungal medications. Topical treatment durations for PV can span from a few days up to 4 weeks. Oral medications are utilised as a second-line treatment for extensive, severe, unresponsive, or recurrent disease.

Three main drug classes are topically used in PV: imidazoles, allylamines and ciclopirox olamine [4].

Imidazoles exhibit a fungistatic action via the inhibition of lanosterol 14-α-demethylase in the ergosterol synthesis pathway [4,81]. That compound is a key element of the fungal cell membrane. Ketoconazole is a highly lipophilic antifungal that has been shown to have an additional antiandrogenic and anti-inflammatory action, and can normalise corneocyte proliferation [4]. It is typically administered as a cream applied twice daily for a 15-day period [1,4]. In a large meta-analysis, its efficacy in PV ranged from 71 to 89% [82]. A combination of ketoconazole 2% cream and adapalene 1% gel has been shown to be superior to ketoconazole formulation alone [83,84]. Other topical azole antifungals can be used every night for 2 weeks [85]. 

Allylamines act via ergosterol synthesis inhibition at the squalene epoxidase level, which results in the simultaneous intracellular toxicity of accumulated squalene and the fungistatic loss of ergosterol [85]. Terbinafine, butenafine, and naftifine were shown to be effective in PV [86,87,88,89,90,91,92,93,94]. A 1% topical terbinafine is usually applied twice per day for 7 days, but the treatment can be extended up to 4 weeks [6]. Importantly, a number of *M. furfur* strains may be resistant to 1% concentration [1,21]. A 1% butenafine is usually used twice per day for 2 weeks [91], whereas 1% naftifine solution was used once per day for 6 days in one uncontrolled study in PV [94].

Ciclopirox olamine is a hydroxypyridone derivative, being a broad-spectrum antifungal, antibacterial, and anti-inflammatory agent [95]. It acts by chelating polyvalent cations (such as aluminium and iron) that inhibits metal-dependent enzymes responsible for peroxides’ degradation in fungal cells [95,96]. The drug also modulates the activity of cytochromes and catalase, affects mitochondrial transport processes, interrupts energy production, and affects cell membrane permeability that impairs the transmembrane transport of nutrients [95]. It is also speculated to be involved in the impairment of DNA repair mechanisms and mitosis. It was shown to be effective in PV in a number of studies [95,97,98]. The common dosage in PV is twice per day for 2 weeks [95].

Non-specific antifungal topicals (e.g., selenium sulphide 2.5%, zinc pyrithione, propylene glycol, sulphur combined with salicylic acid) can be used to eliminate dead corneocytes and thwart further infection in the stratum corneum [99]. Selenium sulphide lotion should be applied once daily (for 10 min), whereas the treatment duration should be 2 weeks. Resistant cases may require overnight application [100]. The effectiveness of topical tacrolimus 0.03% ointment was comparable to topical clotrimazole in a single-blind randomised clinical trial (50 patients treated twice daily for 3 weeks) [101]. Topical cycloserine (transaminase 1 inhibitor) applied twice per day for 5 consecutive days was shown to clear the hyperpigmented lesions of PV [102].

Managing PV during pregnancy requires topical treatments. Ciclopirox and 1% clotrimazole have been found to be safe and efficacious during pregnancy [103].

A practical problem associated with the use of topical medications is the difficulty in applying the creams on large body surfaces. Spray foams and shampoos are preferred over creams, as the latter are more oily and difficult to apply. Treatment response is excellent in the majority of PV cases treated with topical agents only. Moreover, they have a better safety profile, with a lower incidence of adverse effects (local irritation and contact allergy being the most prevalent, especially with selenium sulphide), fewer drug interactions, and lower costs compared to systemic treatment [4,21]. Usually, the time required for recovery is 2–3 weeks [12].

It is important to emphasise that every topical therapy should also target the scalp, a major reservoir of the fungus.

Considering the oral medications, only azolic drugs play a role. Itraconazole and fluconazole are preferred, whereas oral ketoconazole is no longer recommended, due to its unfavourable safety profile (unacceptably high hepatotoxic potential, risk of adrenal insufficiency, and high interactivity with other drugs) [4,6,21]. There are several dosing schedules with fluconazole (e.g., 300 mg weekly, 2–4 weeks) or itraconazole (e.g., 200 mg daily; 5 to 7 day) [1,4]. Of note, oral terbinafine was shown to be ineffective in PV, as unlike the other systemic drugs, it is not excreted with sweat to the skin surface, while epidermal diffusion does provide an effective fungicidal concentration [104,105]. In cases of resistant or persistent infection, a combination of oral and topical therapy might be considered [4]. After completing the treatment, repigmentation may require several months [12].

Narrow-band ultraviolet phototherapy appears to be a promising alternative as a second- or third-line therapy in PV. In a prospective trial including 30 patients with disseminated PV, who experienced over four disease relapses within the 12-month period, narrow-band ultraviolet-B phototherapy was used on a three-times-weekly regime until complete clearance was achieved, but no longer than until week 8 [106]. Excellent response was observed in 66.7% of patients and mild residual disease was observed in 14% patients. In all patients, the lesions on the trunk, arms, and legs disappeared completely, and in some, a few small patches remained above the arms (residual disease). Improvement in skin lesions was observed in week 4 [106].

## 8. General Recommendations to Limit the Risk of Recurrence

Antifungal treatment promptly eliminates the fungus, and the prognosis is generally good. Nevertheless, untreated PV may persist for years. The recurrence rate is high (up to 80%) [4], with a higher morbidity noted among the individuals with a positive family history of PV [1,8,9]. This fact can be explained by the commensal nature of *Malassezia spp*. Thus, patient education and advice on good personal hygiene are pivotal to achieve long-term remissions. Prolonged prophylaxis with topical agents should be considered in recurrent cases, especially during warmer and more humid months. The whole-body application of antifungal shampoos or soaps with selenium sulphide, zinc pyrithione, ketoconazole, and terbinafine might limit the recurrence rate [107]. Nevertheless, it is worth underlining that such management is still poorly supported with the literature. The patients should be informed that no special diet has been shown to be of aid in PV.

Prophylactic use of oral antifungal agents (viz., itraconazole or fluconazole) remains a practical alternative, especially if topical agents fail to eliminate the infection [1,21]. Such a regimen is supported with the results of a large study where prophylactic administration of itraconazole 200 mg once per month for 6 months provided a significantly higher disease-free rate than placebo (88% vs. 57%) [108].

Additional measures include washing and ironing the clothes, as these can serve as a reservoir for *Malassezia* and be the source of recolonisation [22].

## 9. Conclusions

PV is a common, chronic, and relapsing dermatosis with various clinical manifestations. Although the diagnosis is usually made on the basis of the clinical presentation, the additional diagnostic methods described above might be useful for confirmation and differentiating it from other differential diagnosis, especially in atypical presentations. The early initiation of treatment and prompt identification of recurrence help to avoid complications, especially the social burden of pigmentation disorders, significantly affecting the patients’ quality of life. We hope that by implementing the insights gained from this review, healthcare providers can enhance patient care, improve the outcomes, and contribute to the overall well-being of individuals affected by PV.

## Figures and Tables

**Table 1:** Differential diagnosis of pityriasis versicolor.

Condition	Clinical Characteristics	Dermatoscopy
**Hypopigmented Disorders**		
Pityriasis alba [55,56]	Flat, acquired roundish, asymptomatic hypopigmented macules with a subtle scaling, often located on the facial skin in children and adolescents.	Poorly demarcated hypopigmented areas covered with fine scales (lamellar or branny).
Vitiligo [51,56]	Flat, acquired and persistent, uni- or bilateral, well-demarcated hypopigmented roundish or linear macules.	Well-demarcated, non-scaly, diffuse white areas, usually accompanied with perifollicular hyperpigmentation and leukotrichia.
Idiopathic guttate hypomelanosis [51,56]	Flat, acquired hypo- or depigmented, variably sized roundish or polygonal macules predominantly located on sun-exposed sites and sparing the face.	Numerous well- to poorly defined hypopigmented areas characterised by various shades of white, bordering randomly distributed hyperpigmented reticular lines (cloudy sky-like pattern).
Leprosy (borderline or borderline tuberculoid) [56,57]	Distinct, acquired, circular, erythematous plaques with well-defined edges, forming a saucer-like shape, where the margins slope inside.	Orange-yellow or white structureless areas, with reduced number of hair units/follicular and eccrine ostia (white clods and dots, respectively). Orange clods, and vascular polymorphism (lines serpentine, clods, dots) can be present in some cases.
Progressive macular hypomelanosis [58]	Flat, acquired, symmetrically distributed, coalescing, non-scaly hypopigmented macules affecting the trunk and back.	Disseminated folliculocentric depigmented areas displaying subtle pigmentation of reticular lines. If present, delicate white scales are mainly restricted to skin creases.
Ash leaf macules in tuberous sclerosis [56,59]	Congenital, flat lanceolate depigmented macules present in tuberous sclerosis or isolated.	Poorly demarcated depigmented areas. Subtle background reticular lines are preserved.
Nevus depigmentosus [51,56]	Congenital, asymptomatic flat pale macules with fixed shape, typically present at birth.	Poorly defined hypopigmented area with subtle faded physiological reticular lines of background pigmentation and peripheral islands of normal-coloured skin.
Nevus anemicus [56,60]	Congenital, segmental flat area of depigmentation.	Poorly defined whitish area surrounded with red peripheral zone that often feature linear serpentine vessels.
Extragenital lichen sclerosus [51,56]	Acquired, usually asymptomatic, flat-topped papules or plaques affecting the trunk or/and extremities.	Well-delineated structureless white-yellowish areas. Active lesions feature typical yellowish follicular plugs. Diffuse white scale, polarising-dependent white structures (lines, areas, 4-dotted clods, and rainbow pattern), haemorrhages, dotted, linear serpentine and/or branching vessels can be present.
Hypopigmented mycosis fungoides [56,61]	Acquired, patchy hypopigmented areas.	Poorly defined hypopigmented pink-white areas deprived of physiological background pigmentation of lines reticular.
**Hyperpigmented disorders**		
Confluent and reticulated papillomatosis [56,62]	Acquired, flat, asymptomatic, grey-brown scaly papules coalescing into larger patches arranged in a reticular fashion, commonly affecting the torso.	Subtle/small white scales and brownish clods separated by hypopigmented lines (cobblestone appearance, or gyri and sulci pattern).
Becker’s nevus [56,63]	Congenital, irregularly shaped hyperpigmented macule often accompanied by hypertrichosis, commonly affecting shoulder girdle or upper chest.	Poorly demarcated brown reticular or structureless areas and increased hair density.
Idiopathic eruptive macular hyperpigmentation [64,65]	Disseminated, nonconfluent, and asymptomatic pigmented macules affecting the trunk, neck, and proximal extremities, developing in childhood or adolescence without any background of inflammatory lesions or drug trigger.	Pigmented dots and clods distributed over the accentuated lines reticular (physiological pigmentation) with preservation of normally pigmented skin markings and follicular ostia.

## Data Availability

Not applicable.
